# m-Follow up for zinc adherence by caretakers of children with acute watery diarrhoea: A randomized controlled trial

**DOI:** 10.1371/journal.pdig.0000348

**Published:** 2023-10-03

**Authors:** Fatimah Zahra Karim, Rodrick Kisenge, Karim Manji

**Affiliations:** Department of Paediatrics and Child Health, Muhimbili University of Health and Allied Sciences, Dar-es-Salaam, Tanzania; University of New South Wales, AUSTRALIA

## Abstract

The standard of care for children with acute watery diarrhea (AWD) with no dehydration comprises oral rehydration solution, zinc, and feeding advice. Adherence to zinc therapy may be an issue in the management of acute watery diarrhoea. Mobile phones are used by over 90% of the population in Tanzania, thus good means to improve adherence to prescribed medication and/or attendance to follow-up visits. The objective of this study was to see whether m-follow-up improves adherence rate to zinc therapy, possible reasons for non-adherence, in children with diarrhea.: A randomized controlled trial was carried out in a suburban municipality in Dar-es-Salaam. Block randomization of participants was carried out with a block size of 4 and a 1:1 ratio of intervention: control. The intervention group comprised participants who were to be followed up using text messages and voice calls; the control group was to be followed up in outpatient. The outcome of interest was adherence to the full course of 10 days’ oral zinc, reasons for nonadherence and acceptability. Chi-square was used to compare the categorical variables. δ, the targeted difference in adherence between arms, was pre-set at 20%. The total number of participants were 196, of which 98 participants were enrolled in each arm. Full adherence to the 10-day course of zinc sulphate in children with AWD and no dehydration was 84.1% in the control arm and 89.7% in the m-follow up group (*P* = 0.33). m-follow up significantly improved physical attendance at 14-day clinic visit compared to control group (39.8% vs. 60.2%; *P* = 0.006). Commonest reasons for non-adherence in both groups were related to vomiting (67%). Vomiting at enrolment due to gastroenteritis was significantly associated with vomiting zinc sulphate with RR 2.17 (95% CI 1.24–3.79, *P* = 0.007). The acceptability of m-follow-up was high (99%). In conclusion the idea of m-follow-up was well received by participants who considered it acceptable. In this study, the adherence to Zinc dosing was not significantly different between the intervention and control group, and we consider that for zinc in AWD, counselling alone was good enough to achieve high adherence. The trial was registered with the Pan-African Clinical Trial Registry. Trial number: PACTR201711002737120

## Author summary

m-health or use of mobile phones to follow-up pertinent medical issues such as reminder of dosages and attending follow-up clinics is becoming increasingly common. We studied in the context of achieving adherence and compliance as well as return to follow-up among caretakers of children with acute diarrhoea requiring the use of a 10-14-day dose of oral zinc and returning for follow-up at 14 days. This study shed light of the feasibility, acceptance of this method of follow-up. Definition of term: m-follow up is defined as following the caretaker for reminding the dosage of Zinc for the child using a mobile phone call or message. It is used interchangeably with m-contact, meaning phone contact.

## Introduction

Acute watery diarrhoea (AWD) is diarrhoea that lasts fewer than 14 days and presents without frank blood in stools. Hydration status is classified into three groups: no dehydration, some dehydration and severe dehydration by WHO. Children who are not dehydrated, present with intact mental status, and only one or none of the other criteria (sunken eyes, thirst, reduced skin turgor) [1]. Diarrhoea, defined as three or more loose stools in a 24-hour period, affects all populations across the globe and researchers from Tanzania have reported an annual incidence of 6.1–12.6% in children under age of 5 years [2,3].

AWD with no dehydration is best managed with oral rehydration solution, zinc therapy and feeding advice. Zinc has been shown to reduce volume, frequency and duration of diarrhoea [4–7]. It is recommended that Zinc be continued for a full 10- or 14-day course even after diarrhoea has stopped in order to prevent and/or reduce the severity of future episodes of diarrhoea [8].

Adherence to a full course of 10–14 days therapy of Zinc in children with diarrhoea is therefore important.

Nasrin et al reported a 10-day adherence proportion of 55.8% in Bangladesh [9]. Another study from India found a 14-day adherence of 47.8% [10]. However, in Mali, researchers found a higher adherence of 89% and 64% for 10 and 14 days respectively [11].

In Tanzania, we have no data on what proportion of those prescribed zinc sulphate are adherent to the full course. There had been anecdotal reports of poor adherence due to poor tolerance (vomiting), but the extent to which these side effects occur is undetermined. A trial carried out after this one, found that the adherence as reported by caretaker to be over 80% [12].

mHealth (Health services provided via mobile phone) has been successfully used for following up and delivery of pertinent health information in different parts of the world in various populations, including paediatrics. Studies in Kenya have reported a two-fold increase in adherence for anti-retroviral treatment with text message (SMS) reminders. Likewise caregivers of paediatric patients attending emergency department had an 83.6% follow-up rates when reminded by SMS [13–15].

In Tanzania, evaluations of mHealth as an intervention to improve health-care provision include: The Wired Mothers Initiative in Zanzibar—a cluster-randomised controlled trial enrolling 2550 pregnant women. The interventions included SMS from health care workers as well as voice calls from patients. The initiative proved that the intervention group made significantly more visits to their antenatal clinics, were more likely to have a skilled attendant assist their delivery, and had a lower risk of perinatal mortality [15–17]. Other studies in Tanzania have looked at mHealth in the form of mobile phone apps that aid the HCWs in instituting proper care. One such example is a mobile job app developed by Family Health International-360, D-tree and Pathfinder for community health workers to help them choose an appropriate family planning method for patients in a timely manner. Both the health workers and the clients perceived an improvement in the quality of care offered to them [18]. Another app, developed by Mitchel et al., was used to evaluate assessment and classification of sick children using IMCI, comparing before-after data from HCW who initially used the traditional paper based IMCI and later used a mobile phone based IMCI reference. They found that use of the electronic IMCI led to significantly better adherence to the IMCI protocol [19].

Tanzania has a reasonably high mobile phone penetration (teledensity). A 2021/2022 report from the Tanzania Communications Regulatory Authority reports a teledensity of 91% at the end of 2021 [20]. Given this level of mobile phone use among the general population, communication via mobile phones may be a good way to follow up patients with diarrhoea and improve adherence to zinc sulphate prescribed.

For these reasons, the authors postulated that following up patients with AWD and no dehydration by mobile phone using voice calls and SMS may improve adherence to the complete course of zinc prescribed and also that it may improve attendance at an in-person follow up visit.

## Methods

This was a randomised controlled trial carried out between November 2016 and March 2017. We compared follow up by mobile phone to conventional follow up. m-follow-up, m-health and m-contact is defined in this study as any contact made by telephone for the inquiry and reminder of taking Zinc therapy in the child who presented with acute watery diarrhoea. Block randomisation was used with block size of 4 in a 1:1 ratio of intervention: control. Generation of blocks was done using the randomisation feature on Stats Direct software, version 3.

Participants in the intervention arm were contacted via voice calls on days 1, 5 and 10. They were followed up via SMS on the remaining days during the 10-day period. Participants in the control arm were not contacted via SMS/calls during this period. Calls and SMS followed the general outline of “Hello. How are you? How is (Name of child) doing? Is the child vomiting or having diarrhoea? Has the child been given their due dose of Zinc today?” Calls were made and SMS sent by the primary investigator.

All participants were advised to attend the outpatient department on day 5 if symptoms had not resolved, as recommended by the World Health Organization (WHO). They were also provided with pictorial diaries to record the frequency of stools or vomiting. These picture diaries also formed a source document and were collected by the researcher for analysis of trends. Participants in both groups received a prescription for zinc sulphate tablets as per current WHO guidelines (once daily dose of 10mg of elemental zinc for those under 6 months and 20mg for those 6 months and older for a duration of 10 days). All participants were to be seen on day 14 as part of conventional in person follow-up as recommended and at this point the picture diaries were also collected. These provided further information on use of Zinc and the rates of vomiting follow-up as well as to collect the picture diaries provided to them on enrolment. All participants who did not keep their day 14 appointment, regardless of randomisation arm, were called and asked to come in for a visit or if they had travelled, to send their picture diaries by phone. All participants were provided a Tsh.1000/- phone voucher at enrolment and reimbursed with Tsh.2000/- for transport at physical follow up on day 14. This is barely minimum for transport and cannot be considered coercion.

No blinding occurred due to the way the study was set up; the primary investigator acted as recruiter, handled all other participant interaction including all calls, SMS, and in-person follow-up visits, and also carried out the data analysis. However, the randomization list was stored on a computer at a location different from the enrolment site where all patient with diarrhoea received adherence counselling before assessment of inclusion/exclusion criteria and before enrolment.

Primary Outcome: Adherence to zinc sulphate for 10 days, as defined by taking the full course including repeating doses vomited/regurgitated within 30 minutes of ingestion.

Secondary outcomes: Factors affecting adherence to zinc sulphate, rates of attendance at conventional follow-up visit on day 14, participant preferences for time and means of contact, acceptability, and dependability of m-follow-up.

The sample size was calculated using the formula [21,22,23].


$$=2\;x{\left(\frac{\left(Z_{1-\alpha /2}+Z_{1-\beta }\right)}{\delta }\right)}^{2}x\;p\;x(1-p)$$

Where *n* is the number of participants in one arm, *Z* is the Z value for the confidence level, *α* = .05, *β* = .2, p is the estimated proportion of outcome in a control group, in this case 72.5% (arbitrarily taken as the average of the proportions of 10-day adherence in Mali and Bangladesh), δ is the expected difference in adherence between the 2 groups, estimated at 20% and a 20% loss to follow up was assumed [9,11]. A sample size (N) of 196 participants was obtained [21].

All children under the age of five years presenting to the selected health facilities with complains of diarrhoea were assessed for dehydration and managed according to the WHO guidelines for management of diarrhoea. We enrolled children under the age of five years with acute watery diarrhoea and no dehydration, who were prescribed zinc sulphate on the day of enrolment and whose parent/guardian owned a mobile phone. Children with any comorbidity requiring inpatient treatment and those who were already on Zinc supplementation were excluded. Children were recruited sequentially until the sample size was reached.

Data were collected using standardized questionnaires administered verbally by the investigator. The caregiver was taught how to fill a picture diary documenting motions per day, whether zinc sulphate was given, whether the child vomited, and if so, how long after the administration of zinc sulphate this occurred. These picture diaries, which were filled by all participants, were also used to determine adherence and it was assumed these were accurately filled by all caregivers. The picture diary had information on the use of zinc and recognition of dehydration and danger signs and this simple tool has been used in other setting with good compliance in filling them and utilizing them [12,22,23]. The preference times were collected on enrolment and also during the m-follow-up as well as at 5 and 14 days, in both groups.

Information on acceptability was collected using the questionnaire on day 5 and at day 14. This was done by a questionnaire, and some had a scale of 1–10 which was chosen to indicate acceptance. Refusal to participate was also obtained from participants who refused to join, indicating non-acceptance. The text messages responses were also collected and formed part of analysis.

Data was entered into SPSS v.19 and analysed using the same. Rates and Proportions were compared using the χ^2^ test; P-values of <0.05 were considered significant.

Ethical clearance was obtained from the Muhimbili University of Health and Allied Sciences Institutional Review Board and the ethical committee at Temeke Municipal Council. Written informed consent was obtained from the parent/guardian of the child. All participants received the standard management for AWD including counselling for adherence to the zinc. Any other comorbidities diagnosed were treated. In event of complications after enrolment, appropriate management, including a visit to a nearby health facility for standard management was advised. Participants were free to withdraw from the study at any time, with no effect on the care rendered to them.

## Results

**Fig 1:**
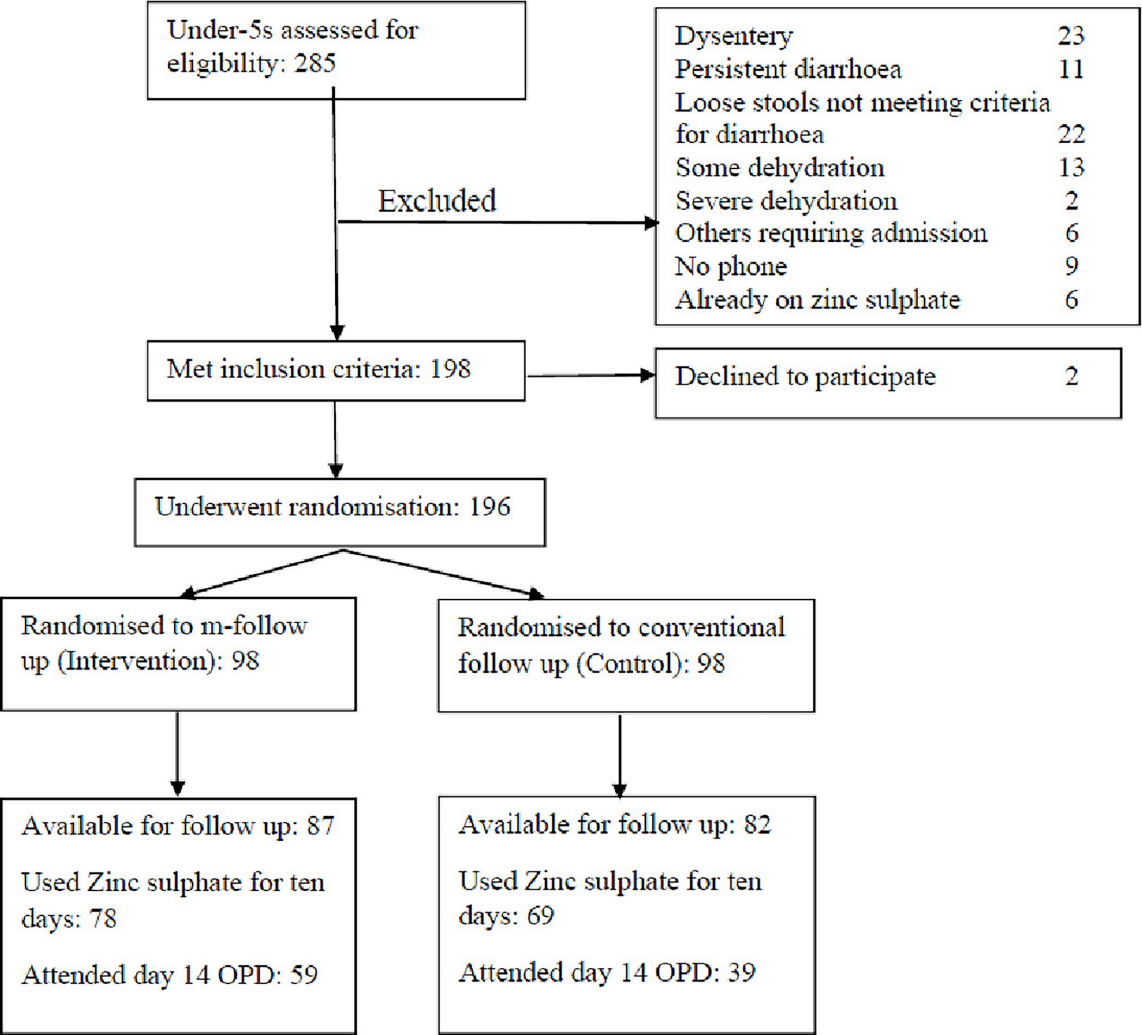
Flow chart of patients assessed, excluded and randomized.

A total of 285 children under the age of five years presented complaining of loose stools during the period of study. Those meeting inclusion criteria were enrolled. The participants’ serial number was then checked against a computer generated randomisation list and the study arm allocated in this way. (Fig 1)

The participants in the two arms were comparable between the two groups in any baseline characteristics, showing effective randomisation. (Table 1)

**Table 1:** Characteristics of children under the age of five years with AWD and no dehydration, and their caregivers.

Characteristics	m-follow up group (Intervention) n = 98	Conventional follow-up (Control) = 98
Age (Mean, Range) months	17.22 (3–52)	17.05 (3–58)
Sex (M)	60 (61.2%)	55(56.1%)
Duration of diarrhoea at enrolment (≤3 days)	89 (90.8%)	85(86.7%)
Parent’s/guardian’s educational level: (N = 95 and 91) None Completed Primary school. Completed O’-levels. Higher Education	7 (7.4%) 49 (51.6%) 34 (35.8%) 5 (5.3%)	2 (2.2%) 54 (59.3%) 33 (36.3%) 2 (2.2%)
Vomiting at baseline (n = 97 and 96)	43 (44.3%)	40 (41.7%)

There was no significant difference in zinc adherence between the patients randomised to m-follow up (Table 2). This lack of significant difference remained even when classifying the participants lost to follow up as non-adherent.

**Table 2:** Adherence among those who returned to clinic to full 10-day course of prescribed Zinc by arm at day 14.

	m-Follow up (Intervention) (n = 87)	Conventional follow up (Control) (n = 82)
Adherent	78 (89.7%)	69 (84.1%)
Non-adherent	9 (10.3%)	13 (15.9%)

p = 0.332 * the number is 169, and this is because 169/196 attended the 14 day in-person visit at the clinic.

The participants were called at day 5 if they were in the m-health arm, and some came to visit the outpatient. Those who were not in the m-health arm, only few came, as indicated, they may have recovered so they find no need to return. Outpatient department attendance on day 14 differed significantly by arm (Table 3). Overall follow-up rate (for all participants collectively) was 82.6% after targeted m-follow up of all patients, regardless of study arm, who did not keep their day 14 appointment with 169 of the 196 subjects being followed up. At day 5, only 98 (50%) were available for follow up, meaning that m-follow up beyond day 5, and up to day 14 increased the overall follow up rate by about 32%, which is a 64% (increase over baseline. This increase was statistically significant (*P* < .001).

**Table 3:** Adherence to in-person follow-up appointment on day 14 by arm.

Attended follow-up visit	m-Follow up (n = 98)	Conventional follow up (n = 98)
Yes	59(60.2%)	39(39.8%)
No	39 (39.8%)	59(60.2%)

p = 0.006

**Fig 2:**
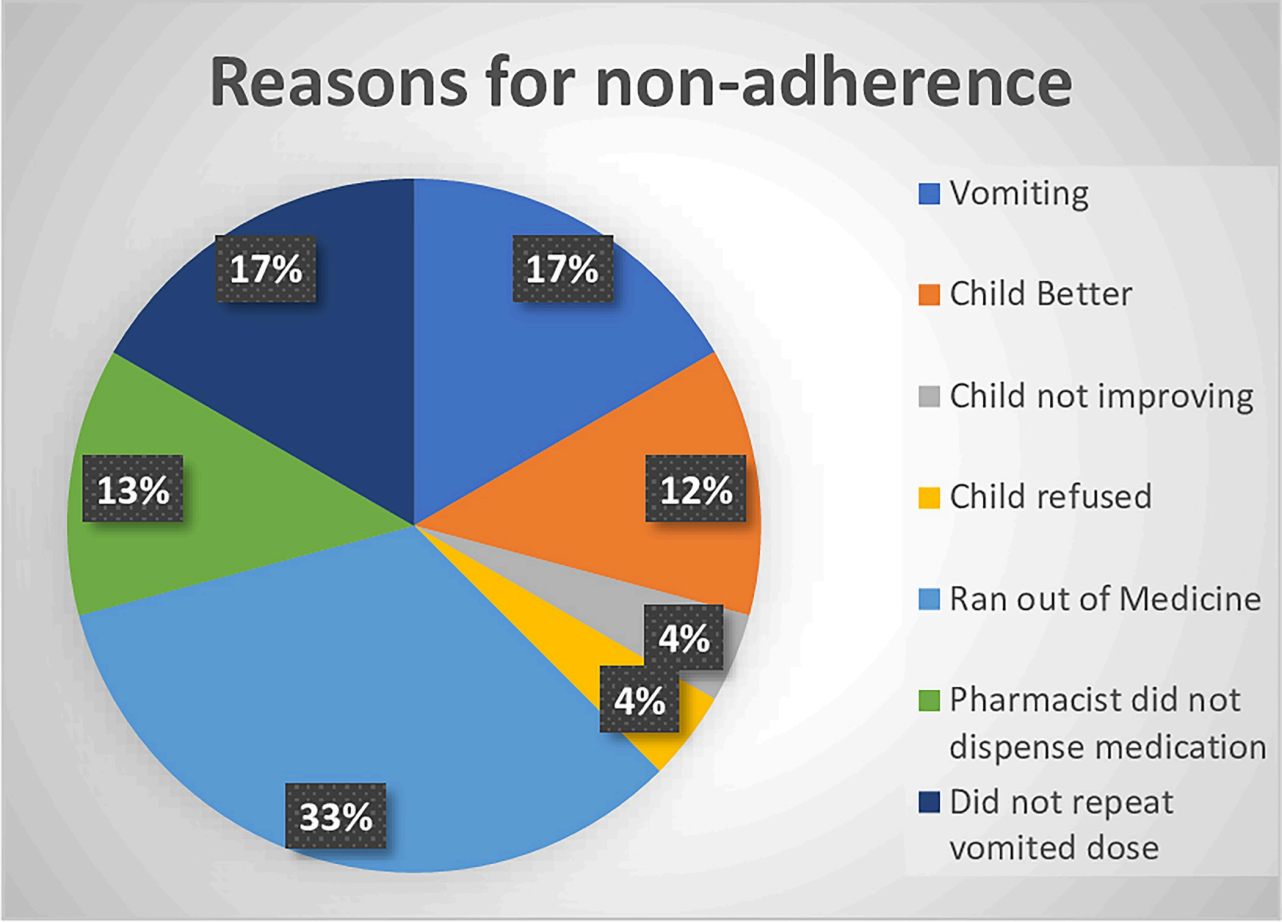
Reasons for non-adherence to zinc.

Reasons for non-adherence to zinc sulphate prescribed were vomiting (17%), not repeating a vomited dose (17%), and running out of medication towards the end because of repeating a vomited dose (33%), not receiving medication from the dispensing pharmacist because of non-availability (13%), child improvement (12%), child refusal (4%) and child not improving (4%). (Fig 2)

It was also noted that those participants who presented with vomiting at baseline were significantly more likely to vomit medication over the course of treatment when compared to those who did not present with this symptom. (Table 4)

**Table 4:** Vomiting at presenting visit may predict future vomiting of medication.

	Vomited medication		
Vomiting at enrolment	Yes	No	Total
Yes	25	45	70
No	15	76	91

RR = 2.17 (95% CI 1.24–3.79) p = 0.007

**Fig 3:**
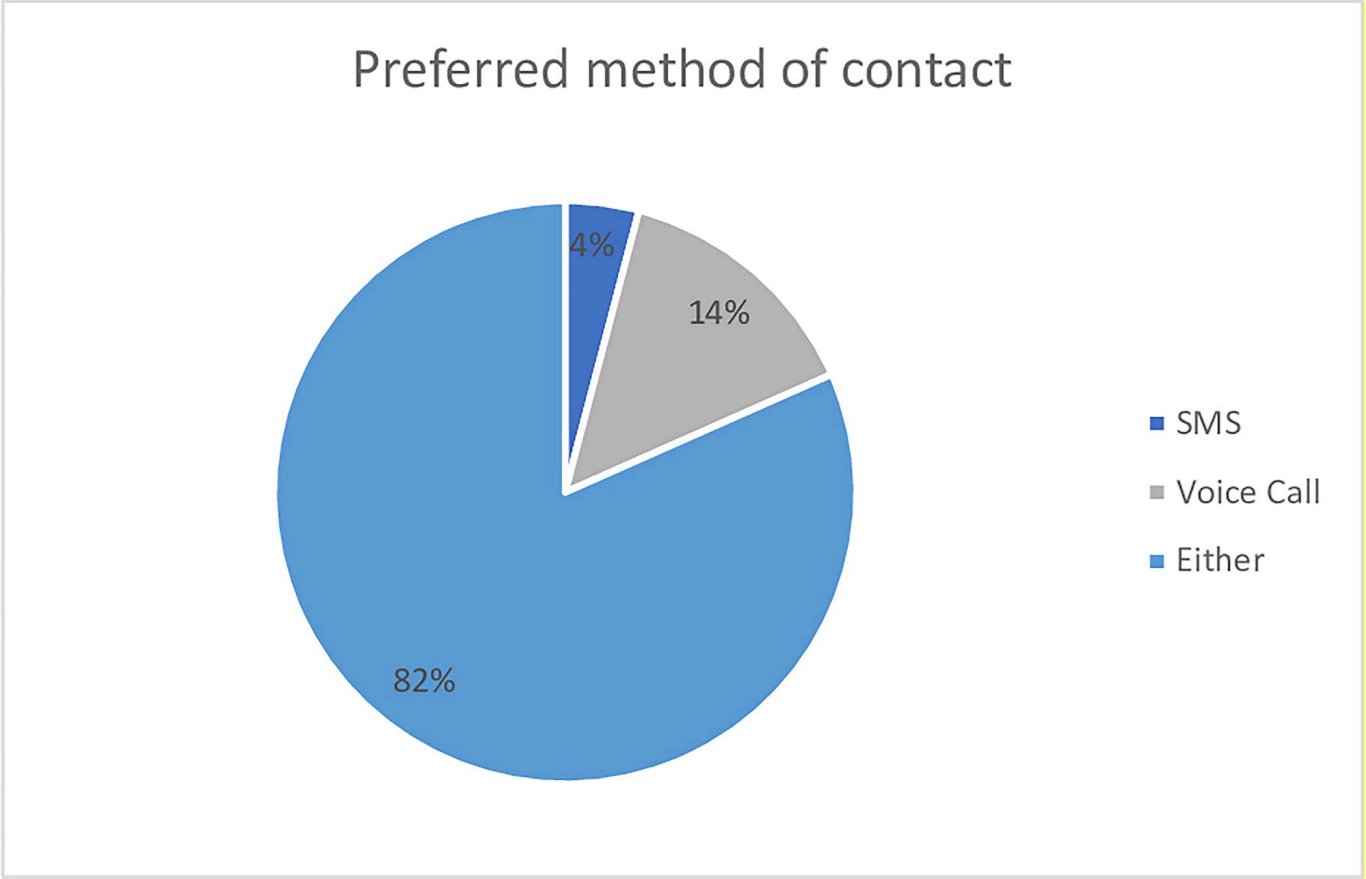
Preferred method of contact.

**Fig 4:**
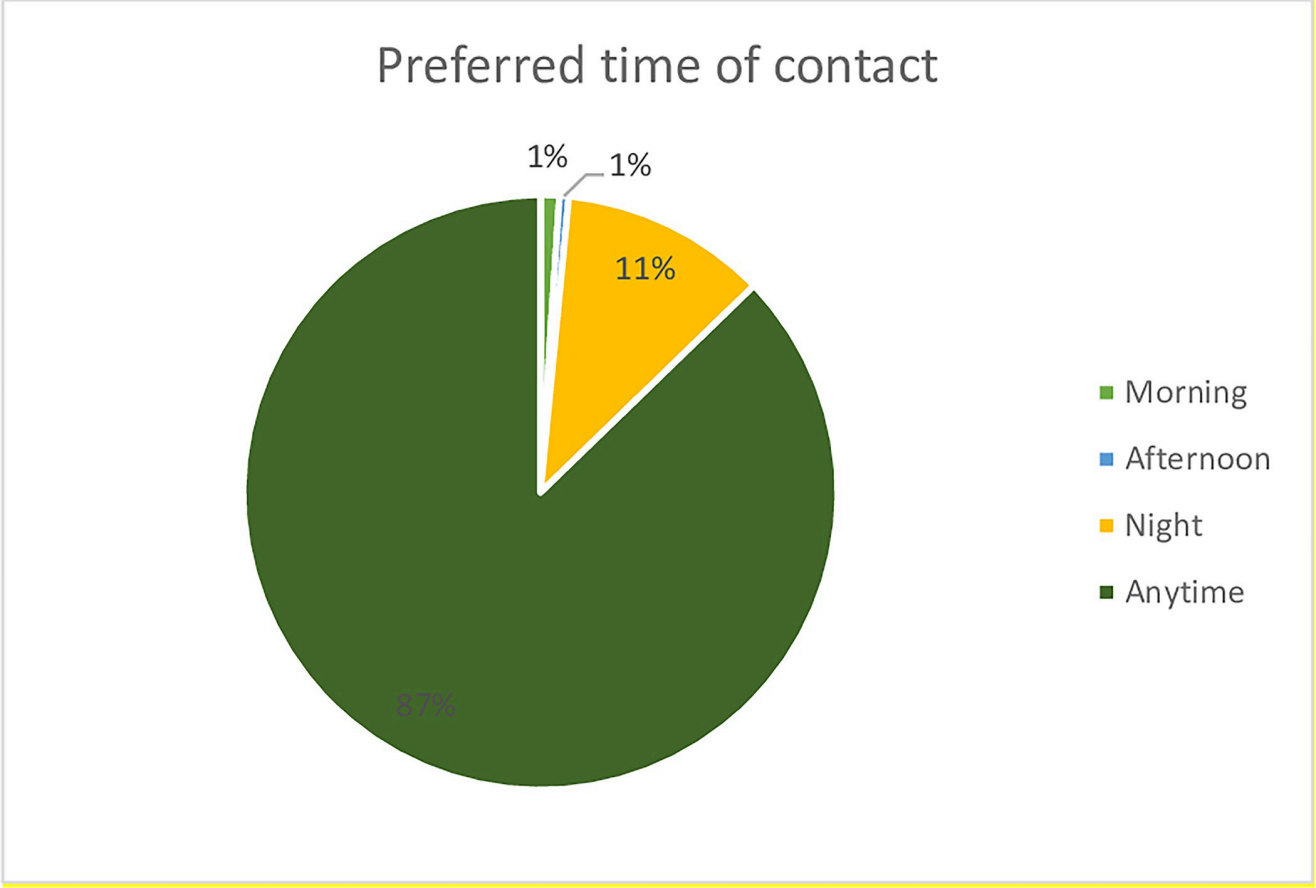
Preferred time of contact.

Of the 196 subjects enrolled, caregivers of 161 (82.1%) had no preference for method of contact, with a minority (8 and 27) preferring SMS (4.1%) and voice calls (13.8%) respectively. (Fig 3) In relation to preferred time, 171 (87.2%) of caregivers had no specific preference for time of contact. 21 preferred to be contacted at night (10.7%), 2 in the morning (1%) and 2 asked to be contacted in the afternoon (1%). (Fig 4)

Of the 98 randomised to m-follow up, 79 (80.6%) responded to at least one SMS, 53 (54.1%) responded to at least half the text messages sent but only 10 (10.2%) responded to all messages. 46.8% initiated some form of contact by mobile phone during the duration of follow up.

## Discussion

Ten-day adherence to prescribed zinc sulphate in children with AWD and no dehydration was high in the control group, which was not followed up by mobile. This was similar to the value found by researchers in Mali [11], but higher than that reported by researchers in Bangladesh [9] and India [10]. This may be because of similarities in the two African populations and differences between the African and Asian populations studied. Studies have shown that children in Africa are less stunted and present with more dehydrating diarrhoea compared to those from Asian sites and that may possibly be one of the reasons. In addition, rotavirus vaccination coverage is much higher in Tanzania and other African countries than in Asia [12]. It may also have been higher in our study participants than in the Asian studies because of the one-to-one effective adherence counselling provided by the recruiter while prescribing the medication.

The desirable adherence for Zinc in diarrhoea to be completed is 100%, a study by Dhingra in Tanzania and India showed a high adherence rate of over 80% in both the Tanzania as well as India site [12]. The 10-day adherence for children followed up daily by mobile phone was slightly higher but this did not reach the targeted increase and was not statistically significant. This may be due to the relatively high adherence in the population at baseline, making a significant increase impossible. Studies which have shown improved adherence to medication with m-contact dealt with chronic illnesses where adherence is a bigger problem, affecting, on average, over half of patients [23–25]. A study on evaluating Smartphone decision-support tool for diarrhoeal disease management in Bangladesh found that this improved adherence to Zinc therapy in children below 5 years age [26].

The three commonest reasons for non-adherence, altogether contributing to two-thirds of non-adherence, were related to vomiting including stopping because of vomiting itself, a vomited dose not being repeated and running out of tablets before day 10 (because of repeating doses which were vomited). This is in contrast to the study from Bangladesh [9] where vomiting accounted for only 12.2% of non-adherence, but where other factors such as the child being cured were more prominent. This may have been prevented by the adherence counselling provided to caregivers at presenting visit, emphasising that a full 10-day course be followed even if the child was feeling better. A study in Ghana indicated that education and counselling of care providers and their patients are key to a successful adherence to treatment in childhood diarrhoea [27].

A few patients did not receive zinc sulphate from the pharmacies they visited. Non-adherence of this form was prevented in the m-follow up arm, since the parent/guardian was informed to alert the study team, who then provided to the missing medication. This is usually not the case under normal circumstances.

Children who presented with vomiting at enrolment were more than twice as likely to vomit their zinc sulphate as those with no vomiting at presenting visit. This points towards baseline nausea and vomiting associated with gastroenteritis playing a role in the vomiting of medication. The zinc sulphate prescribed may further aggravate this nausea and cause the vomiting, or it may play no specific role [12].

Of those meeting inclusion criteria, only two patients declined to participate in the study, showing the wide acceptability of m-follow up in our setting. No specific reasons were given for declining.

Of those randomised to m-follow up, almost half initiated some form of contact (SMS or phone call) during the duration of follow up, usually to ask for medical advice due to a change in the child’s condition such as development of dysentery or dehydration. This shows that many patients, given the opportunity, would seek healthcare/advice in a timely manner, which indicates that mHealth may improve health-seeking behaviour in those who are deterred by the cost of travelling to a health-care facility and/or time spent queuing to see a health-care provider. We also noted that response rates in our study were higher with calls than text messages indicating that voice calls may be a more dependable method of contact [15,19,25].

This study was done in an urban setting where most of those assessed for eligibility owned a mobile phone, confirming that the teledensity in the study population was higher than the national average. In addition, we only enrolled children with AWD and no dehydration; therefore, our results may not be generalizable to all children with diarrhoea with some or severe dehydration. The adherence rate achieved by adherence counselling alone was high enough that it became impossible to achieve the targeted increase in adherence given the number of participants enrolled.

At the onset of the study, while administering the consent form, one has to explain that there will be two groups, one which gets a m-health message follow-up to remind the adherence. The second group will be those who will be followed as usual for 14 days and check for adherence. This according to our interpretation already behaves like an incentivized intervention. Even the control group is aware that their peers will have an SMS reminder. This fact behaves like the incentive to adhere. Since the adherence outcome between the two groups did not differ, the authors found no need to analyze the differences or look for factors improving non-adherence. This is similar to the pilot study by MacCarthy S et al in their study coined SITA in Uganda, although it was for the ART adherence among youth [28]. With the current trend or m-health, we find that if the mother is prompted by a reminder using m-health technology, it may improve the adherence, however, even without this sms prompt, if at the onset, a care-giver is counselled sufficiently on the importance of Zinc in childhood diarrhoea, the desired adherence can be obtained. Using the denominator of all screened and eligible, who did not consent as a proxy to acceptance, we found that the acceptance rate was very high.

In conclusion, m-follow up did not significantly improve adherence but it significantly improved physical attendance at 14-day clinic visit. This study showed that m-follow up is acceptable and that it is potentially a viable option for improving attendance to follow-up visits.

## Supporting information

S1 CONSORT Checklist CONSORT Checklist. (DOC)

S1 Protocol Dissertation Protocol including the picture diary. (PDF)

S1 Data Data file accessible by SPSS software. (SAV)
