# Supplementary material for: m-Follow up for zinc adherence by caretakers of children with acute watery diarrhoea: A randomized controlled trial
Source: PLOS Digit Health. 2023 Oct 3;2(10):e0000348. doi: 10.1371/journal.pdig.0000348 (PMC10547181; doi:10.1371/journal.pdig.0000348)
Supplement: S1 Protocol — (PDF) [file pdig.0000348.s002.pdf]

MUHIMBILI UNIVERSITY OF HEALTH AND ALLIED SCIENCES

SCHOOL OF MEDICINE

DEPARTMENT OF PAEDIATRICS AND CHILD HEALTH

DISSERTATION REPORT

JUNE 2017

ZINC ADHERENCE: A FOLLOW-UP STUDY OF UNDER-FIVES WITH ACUTE WATERY DIARRHOEA USING MOBILE PHONES – A RANDOMISED CONTROLLED TRIAL

MMED CANDIDATE: **DR. FATIMAH ZAHRA KARIM**

HD/MUH/T.04/2014

SUPERVISOR: DR. RODRICK KISENGE

CO-SUPERVISOR: PROF. KARIM MANJI

ZINC ADHERENCE: A FOLLOW-UP STUDY OF UNDER-FIVES WITH ACUTE WATERY DIARRHOEA USING MOBILE PHONES – A RANDOMISED CONTROLLED TRIAL

By Dr. Fatimah Zahra Karim

A Dissertation Submitted in Partial Fulfillment of the Requirements for the Degree of Master of Medicine (Paediatrics and Child Health) of the Muhimbili University of Health and Allied Sciences

Muhimbili University of Health and Allied Sciences
June, 2017

CERTIFICATION

The undersigned certify that they have read and hereby recommend for examination by Muhimbili University of Health and Allied Sciences a dissertation titled: **‘‘Zinc Adherence: A Follow-Up Study Of Under-Fives With Acute Watery Diarrhoea Using Mobile Phones – A Randomised Controlled Trial’’** in partial fulfillment of the requirements for the degree of Master of Medicine (Paediatrics and Child Health) of the Muhimbili University of Health and Allied Sciences.

____

Dr. R. Kisenge

(Supervisor 1)

Date: _________________________

____

Prof. K. P. Manji

(Supervisor 2)

Date: _________________________

DECLARATION

I, Fatimah Zahra Karim, hereby declare that this dissertation is my own original work and that it has not been presented and shall not be presented to any other university for a similar or any other degree award.

Signature…………………………………… **Date**………………………………

STATEMENT OF COPYRIGHT

This dissertation is a copyright material protected under the Berne Convention, the Copyright Act 1999 and other international and national enactments, in that behalf, on intellectual property. It may not be reproduced by any means, in full or in part, except for short extracts in fair dealing, for research or private study, critical scholarly review or discourse with an acknowledgement, without the written permission of the Directorate of Postgraduate Studies, on behalf of both the author and the Muhimbili University of Health and Allied Sciences.

ACKNOWLEDGEMENTS

I would like to acknowledge my supervisors who were supportive throughout this journey. I am also grateful to other faculty members at the department who did not hesitate to extend a helping hand whenever one was needed.

I would also like to thank the DMO of Temeke Municipality and the Medical Officers in charge of the facility used as a recruiting center for making me feel welcome in their jurisdictions.

Lastly, I would like to thank the children and caregivers who participated in this study. This work would definitely not have been possible without their cooperation.

DEDICATION

To my parents, who always believe in me and push me to be the best I can, and to Jafar, who is my pillar of support at all times.

ABSTRACT

BACKGROUND

Diarrhoea is one of the leading causes of illness and death in Tanzanian children under the age of five years. Prevention and treatment of dehydration by the use of ORS (Oral Rehydration Salts) solution can avert most of the deaths caused by diarrhoea but it does not affect the course of the illness. Zinc sulphate has been found to reduce stool output in children with diarrhoea and also shortens the duration of illness. Variable adherence rates have been found in different populations. Mobile phone technology offers a convenient way to follow up these patients to determine adherence. However, mobile-follow up (m-follow up) has been shown to affect outcomes, including adherence to medication, favourably.

OBJECTIVES

To compare adherence to a 10-day course of zinc sulphate in children under five years with acute watery diarrhoea followed up conventionally against those followed up via mobile phones.

METHODOLOGY

A randomised controlled trial was **carried out at Mbagala Zakhem Health Centre in Temeke municipality in Dar es Salaam region**, using block randomisation with block size of 4 in a 1:1 ratio. Caregivers of participants in the intervention group were contacted via text message/voice call daily to remind them asking about the progress/well-being of the child as well as if that day's medication had been taken. A phone call was made on day 1 for introduction, day 5 to inquire about the progress of the child and day 10 to conclude. Participants in the control group were not contacted during this period. All participants were seen at the end of 14 days for conventional follow up and to collect the filled forms. All those who did not attend the follow-up clinic were called for follow-up. The main outcome of interest was the proportions of participants who were fully adherent to the 10-day course of zinc sulphate prescribed. Chi-square and ANOVA were used to compare the categorical and non-normally distributed continuous variables respectively; A p-value of <0.05 was considered to be significant. δ, the targeted difference in adherence between arms, was pre-set at 20%.

RESULTS

98 participants were enrolled in each arm. Subjects were serially enrolled and randomised into one of two arms. Full adherence to the 10-day course of zinc sulphate in children with AWD and no dehydration was 84.1% in those only followed up conventionally and 89.7% (p=0.33) in the m-follow up group. m-follow up significantly improved attendance at 14-day conventional clinic visit for physical follow up (39.8% vs 60.2%; p=0.006). Commonest reasons for non-adherence in both groups were related to vomiting (67%). Vomiting at enrolment was significantly associated with vomiting zinc sulphate with RR 2.17 (95% CI 1.24-3.79, p=0.007). m-follow up was found to be largely acceptable and dependable in our setting.

CONCLUSION

m-follow up did not significantly improve 10-day adherence to zinc sulphate; adherence counselling alone was adequate in producing high adherence rates. Two-thirds of non-adherence was related to vomiting of medication, which was significantly associated with vomiting at presenting visit and should therefore be taken into account when prescribing/dispensing medication as well as planning follow-up. m-follow up, which is both acceptable and dependable, improved attendance at conventional clinic visit and may be used as a strategy for the same.

TABLE OF CONTENTS

CERTIFICATION ii
DECLARATION iii
STATEMENT OF COPYRIGHT iii
ACKNOWLEDGEMENTS iv
DEDICATION v
ABSTRACT vi
LIST OF FIGURES ix
LIST OF TABLES x
DEFINITION OF KEY TERMS xi
LIST OF ABBREVIATIONS USED xii
1.0 INTRODUCTION 1
2.0 LITERATURE REVIEW 3
3.0 CONCEPTUAL FRAMEWORK 6
4.0 PROBLEM STATEMENT 6
5.0 RATIONALE 7
6.0 RESEARCH QUESTIONS 7
7.0 HYPOTHESES 7
8.0 OBJECTIVES 7
9.0 METHODOLOGY 8
10.0 ETHICAL CONSIDERATIONS 11
11.0 RESULTS 13
12.0 DISCUSSION 17
12.1 STRENGTHS 19
12.2 LIMITATIONS 19
13.0 CONCLUSION 19
14.0 RECOMMENDATIONS 20
15.0 REFERENCES 21
16.0 APPENDICES 26
16.1 APPENDIX 1: QUESTIONNAIRE 26
16.2 APPENDIX 2: PICTURE DIARY 28
16.3 APPENDIX 3: CONSENT FORM 30

LIST OF FIGURES

Figure 1: Conceptual Framework.. 6

Figure 2: Flowchart of Patients Assessed, Excluded and Randomised. 13

Figure 3: Reasons for non-adherence to zinc sulphate 15

Figure 4: Preferred method of contact 16

Figure 5: Preferred time of contact.. 16

LIST OF TABLES

Table 1: Characteristics of children under the age of five years with AWD and no dehydration, and their caregivers 14

Table 2: Outcomes by arm.. 15

DEFINITION OF KEY TERMS

Diarrhoea: The passage of loose stools, three or more times a day

Acute watery diarrhoea: Diarrhoea not containing blood and lasting less than 14 days

ehealth: The cost-effective and secure use of ICT in support of health and health-related fields, including healthcare services; health surveillance; health literature; and health education, knowledge, and research

mhealth: A type of eHealth service where mobile phone technology is utilized in health care/information delivery

m-follow up: Follow up of patients by the use of mobile phones

Teledensity: The number of phones owned per 100 people in the population. This is represented by a percentage.

LIST OF ABBREVIATIONS USED

ART	Anti-Retroviral Therapy
AWD	Acute Watery Diarrhoea
ED	Emergency Department
eHealth	Use of electronic interventions in health care
IRB	Institutional Review Board
HCW	Health Care Worker
HIV	Human Immunodeficiency Virus
m-follow up	Follow up by use of mobile phones
mHealth	Provision of health services via mobile phones
MUHAS	Muhimbili University of Health and Allied Sciences
ORS	Oral Rehydration Salts
OPD	Out-Patient Department
SMS	Short Message Service
WHO	World Health Organization

1.0 INTRODUCTION

Diarrhoea is defined as the passage of three or more loose stools over a period of 24 hours. It may be classified into three categories: acute watery diarrhoea, which is not blood stained and lasts less than 14 days, dysentery, where stools are mixed with blood and persistent diarrhoea, which lasts more than 14 days (1).

Magnitude of the problem

Diarrhoea affects all populations across the globe, but children under the age of five years, especially those living in developing countries are most vulnerable. Factors contributing to this include shortage of clean drinking water, poor hand hygiene and improper disposal of wastes. Each year, there are about 1.7 billion cases of diarrhoea worldwide. (1)

Globally, acute watery diarrhoea in children under the age of five years is most commonly of viral aetiology, with the commonest cause being the rotavirus. This is also true in Tanzania (2,3). This being the case, antibiotics are ineffective in managing these patients, and when prescribed, only add to the globally growing burden of antibiotic resistance (4,5).

Globally, diarrhoea causes 760,000 deaths in children under the age of five years annually, making it the second most common cause of death in children in that age group (1). World Health Organization (WHO) estimates that in developing countries, children under the age of 3 years will suffer an average of 3 episodes of diarrhoea per year (1). The mortality rate from diarrhoea varies by country. An analysis of data from 7 developing countries reported cause-specific mortality rates varying from 2.3% to 29.5% (6).

Cross-sectional surveys in Tanzania have reported that 6.1 – 12.6% of children under 5 years are afflicted by the disease at any one time (7,8). Diarrhoea was found to cause 2.3% of deaths in children under the age of five years (6). In 2014, 46,332 children under-5 suffered from diarrhoea in Dar es Salaam region. Of these, about 10% had no dehydration, 72% received zinc, and 92% received Oral Rehydration Salts (ORS) (Principal Secretary, MIS/Ministry of Health and Social welfare. 2015).

History of Management of Acute Watery Diarrhoea

Diarrhoea causes dehydration and electrolyte imbalance, which cause death when not properly managed. A historical review of the then available literature demonstrated how rehydration with different parenteral and enteral fluids led to a drop in mortality associated with childhood diarrhoea. (10)

In 1978, WHO recommended an ORS to prevent and treat dehydration and electrolyte imbalance caused by diarrhoea. Over the years, the search for a better formulation continued and in 2002, WHO introduced a new formula for ORS with lower osmolarity, which was proven to rehydrate better with no associated increase in diarrhoea or vomiting that had been noted with the older formulation(11). Proper use of this ORS prevents and treats dehydration and hence prevents deaths but does little to alter the duration, frequency or stool output during a diarrhoeal episode. This may lead to irrational use of other drugs including antibiotics and anti-motility agents either by the caregiver or the health care worker (HCW) in an effort to alter the course of illness (12).

Treatment with zinc during episodes of diarrhoea has been shown to reduce stool output and duration of diarrhoea under-5s living in developing countries (13,14). It does this by improving the absorption of water and electrolyte, inducing regeneration of the intestinal mucosa including increasing the levels of brush border enzymes to prevent further loss of fluids and electrolytes, and enhances the immune response (15). Zinc treatment for acute watery diarrhoea for the recommended 10-14 days has also been shown to prevent repeated episodes of the same over the next 2-3 months (16). Zinc has also repeatedly been demonstrated to reduce incidence of diarrhoea among well children when used as a supplement. (17–19)

Several studies comparing different zinc salts found no difference in efficacy between different salts. Based on this, WHO recommends zinc sulphate as the supplement of choice, given its lower cost. (20)

mHealth in Tanzania

Tanzania National eHealth Strategy (2012-2018), released in 2013 by the Ministry of Health and Social Welfare, defines eHealth as "the cost-effective and secure use of ICT in support of health and health-related fields, including healthcare services; health surveillance; health literature; and health education, knowledge, and research." It further specifies that mHealth is a type of eHealth service where mobile phone technology is utilized in health care/information delivery. Strategic Objective 7 is listed as "Enable electronic delivery and interventions of health services in order to reduce child mortality…" (21) Tanzania has a reasonably high mobile phone penetration (teledensity). A 2015 report from the Tanzania Communications Regulatory Authority reports a teledensity of 79% at the end of 2015. (21) Given this level of mobile phone use among the general population, communication via mobile phones may be a good way to follow up patients in Tanzania.

2.0 LITERATURE REVIEW

Zinc adherence

One of the known adverse effects of zinc is that it may induce vomiting.(13,18) When it does, it is usually a single episode, within 30 minutes of taking zinc.(18) This may limit adherence to the full course, as well as efforts to rehydrate/prevent dehydration in children since vomiting may hinder the use of ORS.

Several community-based studies have shown that adherence to zinc for the full duration of 10-14 days is less than satisfactory. One study from Bangladesh reported a 10-day adherence proportion of 55.8%(22) whereas one from India found a 14-day adherence of 47.8%(23). However, in Mali, researchers found a higher adherence of 89% and 64% for 10 and 14 days respectively.(24) Reasons for poor adherence included the fact that the child was better, vomiting, refusing tablets, not better, or that the caregiver forgot.(22,24)

Use of SMS/voice calls for follow up of patients

mHealth has been successfully used for follow up and delivery of pertinent health information in different parts of the world in various populations, including paediatrics. Examples include a 2008 study in San Francisco, California that showed that sedentary

women were more motivated to increase their physical activity when prompted by a message on their mobiles(25). A 2012 review of 14 papers revealed that SMS may be a good intervention medium for weight loss in obese adults(26). In 2012, a systematic review of 13 studies that used electronic reminders to improve adherence to chronic medication was published. The reviewers found evidence that in 3 of the 4 studies using SMS as the method of reminder, the intervention was found to improve adherence over a 6 month period(27).

A study carried out in the US in 1993, randomised paediatric emergency department (ED) patients to intervention or control groups. Parents of those assigned to the intervention group received a phone call 12-30 hours after discharge to remind them of discharge instructions. Participants in the intervention group were 1.5 times more likely to follow up with their primary physician as advised, however similar proportions of both groups reported to filling the prescription provided and following other discharge instructions(28). A Chinese study reported significantly improved Out-Patient Department (OPD) attendance in paediatric cataract patients whose parents received mobile SMS reminders(29). Another study from Argentina enrolled new mothers who had tested positive for Chagas disease in order to physically follow up the mother-child pair. SMS was used to remind the women about their follow-up visit. About 89% of women agreed to be contacted by SMS, and of these, 91% replied to the SMS and 92% said the text message was a useful reminder.(30)

In East Africa, researchers reported that they found HIV positive Kenyans initiated on ART were more than twice as likely to achieve a 90% adherence rate if they were randomised to a group assigned to receive weekly SMS reminders over a period of 48 weeks compared to a control group that received no such reminders(31). Another study in Kenya found that 89.3% of families presenting to the paediatric ED had access to mobile phones and a successful follow-up was made in 83.6% of these cases(32).

In Tanzania, evaluations of mHealth as an intervention to improve health-care provision include The Wired Mothers Initiative in Zanzibar was a cluster-randomised controlled trial, which enrolled 2550 pregnant women. The interventions included SMS from HCWs as well as voice calls from patients. This initiative proved that the intervention group made

significantly more visits to their antenatal clinics, were more likely to have a skilled attendant assist their delivery, and had a lower risk of perinatal mortality. (33–35)

Other studies in Tanzania have looked at mHealth in the form of mobile phone apps that aid the HCWs in instituting proper care. One such example is a mobile job app developed by Family Health International-360, D-tree and Pathfinder for community health workers to help them choose an appropriate family planning method for patients in a timely manner. Both the health workers and the clients perceived an improvement in the quality of care offered to them.(36)

Another app, developed by Mitchel et al., was used to evaluate assessment and classification of sick children using IMCI, comparing before-after data from HCW who initially used the traditional paper based IMCI and later used a mobile phone based IMCI reference. They found that use of the electronic IMCI led to significantly better adherence to the IMCI protocol.(37)

Acceptability of mHealth

A study that recruited clients of Totohealth Kenya, carried out in 2015 found that more than 50% of the women have access to the phone at all times of the day, however most (40%) prefer to receive SMSs between 8 and 10am. Seventy-one percent of these women reported interest in messages related to breastfeeding and nutrition and about 50% in prevention of infectious and non-communicable diseases. Forty-two percent preferred to receive SMSs only whereas 39% would have liked to receive voice calls too. Eighty-one percent of them found the information contained in the SMSs very useful whereas the other 19% found it somewhat useful. Fewer than 10% responded to the messages often and 43% never responded to them. Fifty-seven percent of the women said the messages influenced them to attend reproductive and child health clinics and 69% reported they were influenced to visit a health care facility more often. *(Personal communication. F. Karim -- F. Ruiter, Totohealth. Aug 2016)*

3.0 CONCEPTUAL FRAMEWORK

Figure 1: Conceptual Framework

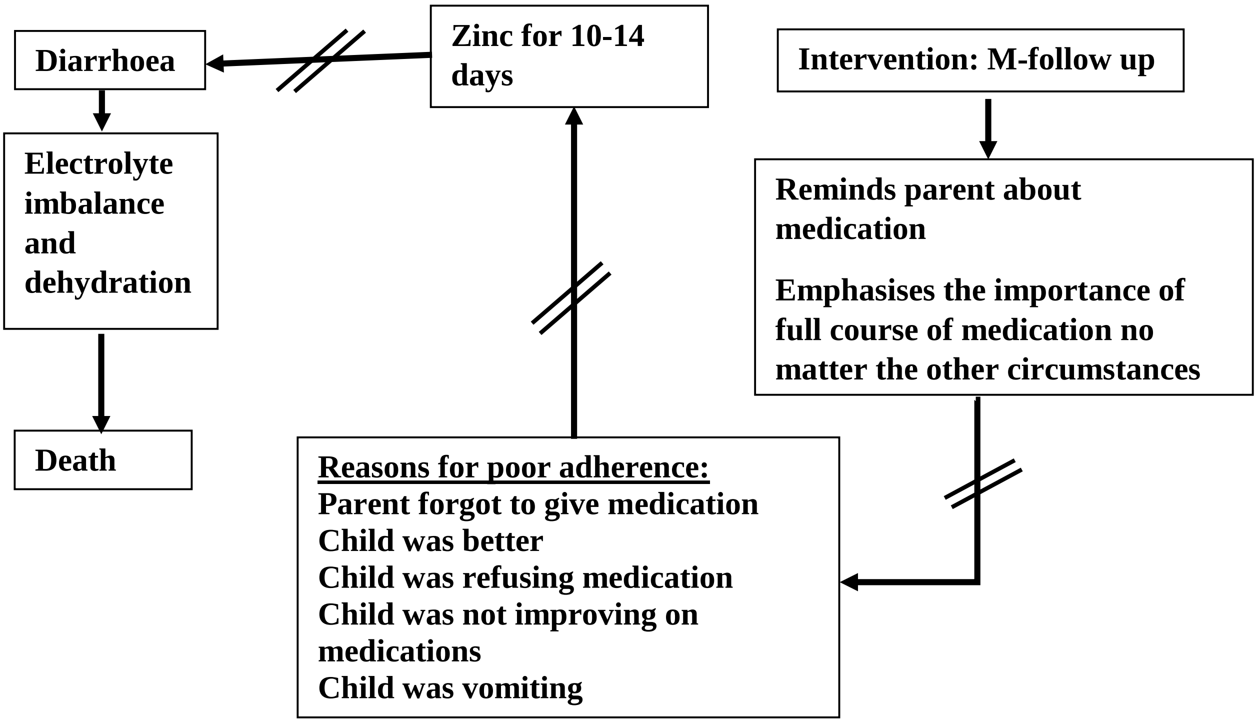

m-follow up would serve to remind parents about medication and emphasize the importance of a full course, hence targeting the reasons known to cause poor adherence. Given this, we would expect the child to use zinc sulphate for the full course, which has been shown to reduce frequency, volume and duration of diarrhoeic stools as well as future incidence of the illness. This may, in turn, reduce morbidity and mortality associated with diarrhoea.

4.0 PROBLEM STATEMENT

In Tanzania, we have no data on what proportion of those prescribed zinc sulphate are adherent to the full course. There have been anecdotal reports of poor adherence due to poor tolerance (vomiting), but the extent to which these side effects occur is also undetermined.

5.0 RATIONALE

Most studies using m-follow up have been done in chronic conditions such as **HIV/AIDS, metabolic syndrome and in pregnancy.** It is not known if m-follow up can affect outcomes in acute illnesses. M-follow up for adherence to prescribed medication has not been objectively studied in Tanzania. If found to be effective, it may help improve outcomes in various conditions by improving adherence.

6.0 RESEARCH QUESTIONS

- What proportion of children with AWD and no dehydration are adherent to a 10-day course of zinc sulphate?
- What are the factors related to non-adherence?
- Is mHealth acceptable and **dependable** in our setting?
- Does use of mHealth as a follow up strategy improve adherence to zinc sulphate in children with AWD and no dehydration?
- Does it improve adherence to clinic appointment?

7.0 HYPOTHESES

Null Hypothesis, H_0: There is no difference in adherence to zinc sulphate between those who are followed up conventionally and those who are followed up by mobile.
Alternative Hypothesis, H_1: Adherence to zinc sulphate is better in the group followed up by mobile phones.

8.0 OBJECTIVES

8.1 Broad Objective

To compare adherence to a 10-day course of zinc sulphate in children under five years with acute watery diarrhoea followed up conventionally against those followed up via mobile phones.

8.2 Specific Objectives

1. To determine the baseline adherence to zinc sulphate in children with AWD and no dehydration when there is no intervention other than adherence counselling.
2. To determine factors related to non-adherence to zinc sulphate in children with AWD and no dehydration.
3. To determine whether there is a difference in adherence to zinc sulphate between the group followed up via mobile phones and that followed up conventionally.
4. To determine whether there is a difference in attendance at 14-day follow-up between the two groups.
5. To ascertain acceptability of m-follow up in children under five years of age who are suffering from acute watery diarrhoea.
6. To determine **dependability** of m-follow up in children under five years of age who are suffering from acute watery diarrhoea.

9.0 METHODOLOGY

9.1 Study design

This was a randomised controlled trial, comparing m-follow up to conventional follow up methods. Block randomisation was used with block size of 4 in a 1:1 ratio of intervention:control. Generation of blocks was done using the randomisation feature on StatsDirect software, version 3.

Participants in the intervention arm were contacted via voice calls on days 1, 5 and 10. They were also followed up via SMS on the remaining days during the 10-day period, namely days 1, 2, 3, 4, 6, 7, 8, and 9. Participants in the control arm were not contacted via SMS/voice calls during this 10-day period. They were seen at a conventional OPD on day 5 if symptoms had not resolved, as is recommended by WHO. **Participants in both groups received a prescription for dispersible zinc sulphate (Ped Zinc) as per current WHO guidelines, that is, a once daily dose of 10mg of elemental zinc for those under 6 months and 20mg for those 6 months and older for a total duration of 10 days.** All participants were to be seen on day 14 as part of conventional follow-up as well as to collect their picture

diaries. All participants who did not keep their 14-day clinic appointment were called and asked to come in for a visit or if they had travelled, to send their picture diaries by phone. This was regardless of what arm they were originally enrolled in. All participants were provided a Tsh.1000/- phone voucher at enrolment and reimbursed with Tsh.2000/- for transport at physical follow up on day 14.

Primary Outcome: Adherence to zinc sulphate for 10 days, as defined by taking full course of prescribed medication including repeating doses vomited/regurgitated within 30 minutes of ingestion.

Secondary outcomes:

- **Factors affecting non-adherence to zinc sulphate**
- **Attendance at conventional follow-up visit on day 14**
- **Participant preferences for time and means of contact**
- **Dependability of mobile phones as means of follow up**

9.2 Study area

Temeke is one of 5 municipalities in Dar es Salaam region in Tanzania. It has an area of 240 km^2 and is populated by 1,205,949 people as of 2012. This number was projected to rise to 1,597,479 by 2017. (38) **The area has two major seasons annually: rainy and dry, with the rainy season divided between the short rainy season from November to January and long, heavy rains which last from March to June each year.** (39)

The informal sector accounts for almost half of the active work force, including agricultural activities. Trade and industry contribute to 60% of the economy. (39)

The health centre where data were collected, Mbagala Zakhem Health Centre caters to the wards surrounding it, namely, Chamazi, Charambe, Kibada, Mbagala Rangi Tatu and Mbagala Kuu. About 100-150 children under the age of five years are seen at the Reproductive and Child Health clinic each weekday. This includes sick and well children. Among these, an average of 0-2 children per day present with diarrhoea during the dry season and 5-7 children per day present with the same during the rainy seasons.

9.3 Population of interest

Children under five years of age with acute watery diarrhoea who were being treated on an outpatient basis.

9.4 Sample size

$$N = 2\,x \left(\frac{(Z_{1-\alpha/2} + Z_{1-\beta})}{\delta}\right)^2 x\,p\,x\,(1-p) \qquad \textbf{(40)}$$

Where N is the number of subjects in one group

Z is the Z value for the confidence level

α is the value of type I error: 0.05 ($Z_{1-\alpha/2}$ = 1.96)

β is the value of type II error: 0.2, ($Z_{1-\beta}$ = 0.845)

P is the estimated proportion of outcome in control group, in this case 72.5% (arbitrarily taken as the average of the prevalence of 10-day adherence in Mali and Bangladesh)

δ is the expected difference in adherence between the 2 groups, estimated at 20%

Therefore, $N = 2\,x \left(\frac{1.96 + 0.845}{0.2}\right)^2 x\,0.725\,x\,0.275$

N= 78.5

Hence, total sample size = N x 2 = 157

Assuming a 20% loss to follow-up, n/(1-0.2)= n/0.8 = 196 study participants.

9.5 Sample selection

All children **under the age of five years** presenting to the selected health facilities in Temeke district with complains of diarrhoea were assessed for dehydration and managed according to the WHO guidelines for management of diarrhoea. Those meeting the inclusion criteria were recruited into the study until the sample size was reached.

- Inclusion criteria
  - ✓ Child **under the age of five years** with:
    - Acute watery diarrhoea
    - No dehydration
    - Prescribed zinc sulphate on the day of enrolment

- ✓ Parent/guardian owns mobile phone
- Exclusion criteria
    - × Any comorbidity requiring admission
    - × Already on day 2 or more of zinc sulphate

9.6 Data Collection

Data were collected using standardized questionnaires (appendix 1) **administered verbally by the investigator in Swahili, documenting participant characteristics such as age, sex, duration of diarrhoea, and preferences for mobile contact such as preferred time and method of contact. The caregiver was also taught how to fill a picture diary (appendix 2). After this understanding was checked by asking the caregiver to explain the process in full.** The picture diary was to be filled by caretaker daily, documenting the progress of the child in terms of motions per day, whether zinc sulphate was given or not, whether the child vomited or not, and if the child vomited, how long after the administration of zinc sulphate this occurred. The picture diaries were also used to determine adherence or lack thereof and it was assumed these were accurately filled by all caregivers.

9.7 Data entry and analysis

Data was entered into SPSS v.19 and analysed using the same. Intention to treat analysis was used. Proportions were compared using the χ^2 test (or Fisher's exact test, when appropriate). Means and ranges were compared for continuous variables. The one continuous variable (patient age) was found to not be normally distributed; therefore, one-way ANOVA was used for comparing the two groups. For all these tests, p-values of <0.05 were considered significant.

10.0 ETHICAL CONSIDERATIONS

Ethical clearance was obtained from MUHAS IRB as well as the ethical committee at Temeke Municipal Council. Written informed consent was obtained from the parent/guardian of the child.

All participants in both arms received the standard management for AWD including counselling for adherence to the zinc. Any other comorbidities detected at presentation were treated.

In event of complications after enrolment, appropriate management, including a visit to a nearby health facility for standard management (when warranted) was advised. Participants were free to withdraw from the study at any time they wished to do so, with no effect on the care rendered to them.

11.0 RESULTS

A total of 285 children under the age of five years presented with complains of loose stool during the period of study. Those meeting inclusion criteria were enrolled and treated. The participants' serial number was then checked against a computer generated randomization list and the study arm allocated in this way. (Figure 2)

Figure 2: Flowchart of Patients Assessed, Excluded and Randomised.

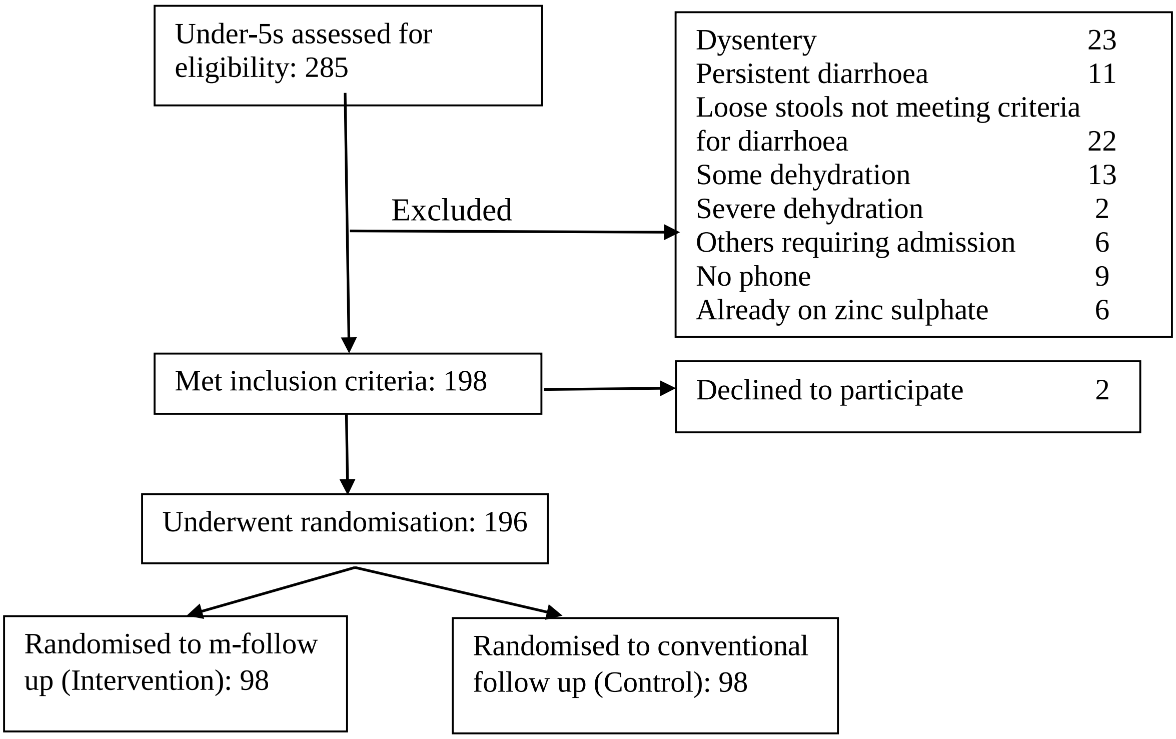

The participants in the two arms were comparable with no significant differences between the two groups in any baseline characteristics, showing effective randomization. (Table 1)

Table 1: Characteristics of children under the age of five years with AWD and no dehydration, and their caregivers

Characteristics	Intervention group (m-follow up) N=98	Control group (Conventional f/up) N=98	p-value
**Age (Mean, Range) months**	17.22 (3-52)	17.05 (3-58)	0.92*
**Sex (M)**	60 (61.2%)	55(56.1%)	0.47**
**Duration of diarrhoea at enrolment (≤3 days)**	89 (90.8%)	85(86.7%)	0.47**
**Parent's/guardian's educational level: (N=95 and 91)**			0.72**
**None**	7 (7.4%)	2 (2.2%)	
**Completed Primary school**	49 (51.6%)	54 (59.3%)	
**Completed O'levels**	34 (35.8%)	33 (36.3%)	
**Higher Education**	5 (5.3%)	2 (2.2%)	
**Vomiting at baseline (N=97 and 96)**	43 (44.3%)	40 (41.7%)	0.83**

*ANOVA ** χ^2

There was no significant difference in zinc adherence between the patients randomized to m-follow up and conventional follow up (p=0.332). (Table 2) This lack of a difference remained even when classifying the participants lost to follow up as non-adherent.

OPD attendance on day 14 was differed significantly by arm, with 60.2% of those randomized to m-follow up attending but only 39.8% of the control arm doing so (p=0.006) (Table 2) Overall follow-up rate was 82.6% after targeted m-follow up of all patients who did not keep their day 14 appointment. Before this targeted follow up, the follow up rate was only 50%, meaning that m-follow up increased the overall follow up rate by about 32%, which is a 64% increase over baseline. This increase was statistically significant (χ^2 46.8, p<0.001).

Table 2: Outcomes by arm

	m-Follow up	Conventional follow up	p-value
**Adherent to Zinc sulphate N= 87 and 82**	78 (89.7%)	69 (84.1%)	0.332
**Attended OPD N= 98 and 98**	59(60.2%)	39(39.8%)	0.006

Various reasons were given for non-adherence to zinc sulphate prescribed, with vomiting related causes being the most common as illustrated in figure 3 below.

Figure 3: Reasons for non-adherence to zinc sulphate

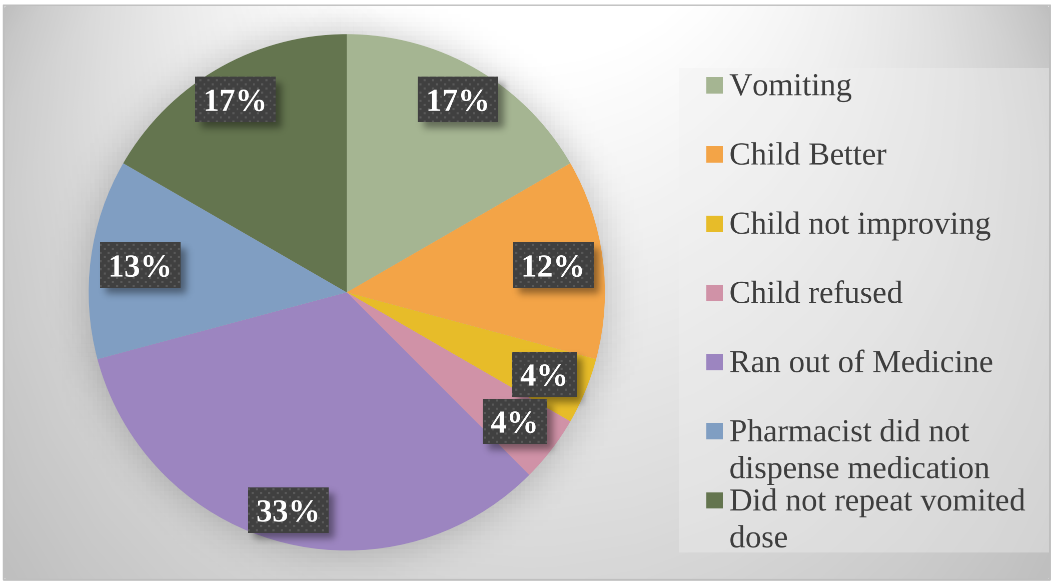

It was also noted that those participants who presented with vomiting at baseline were significantly more likely to vomit medication over the course of treatment when compared to those who did not present with this symptom. (RR 2.17, p=0.007)

Figure 4: Preferred method of contact

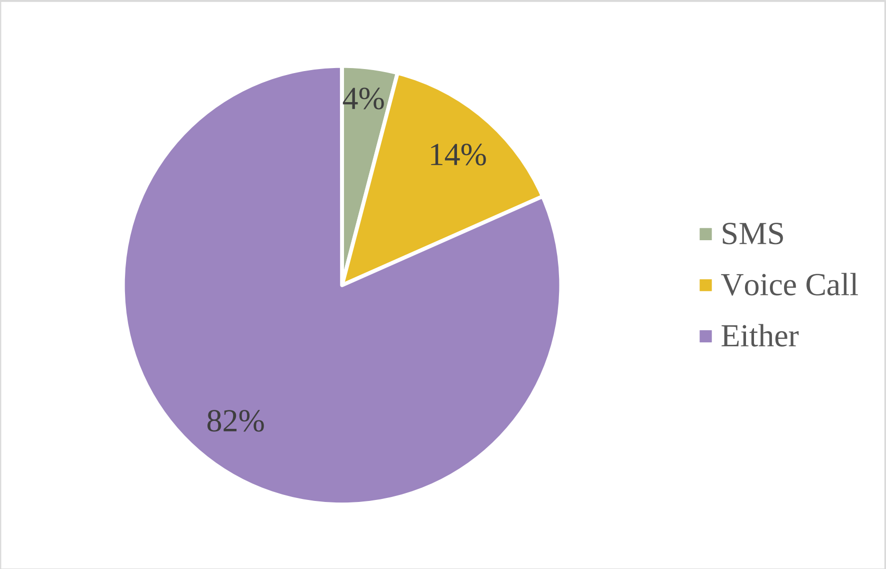

Figure 5: Preferred time of contact

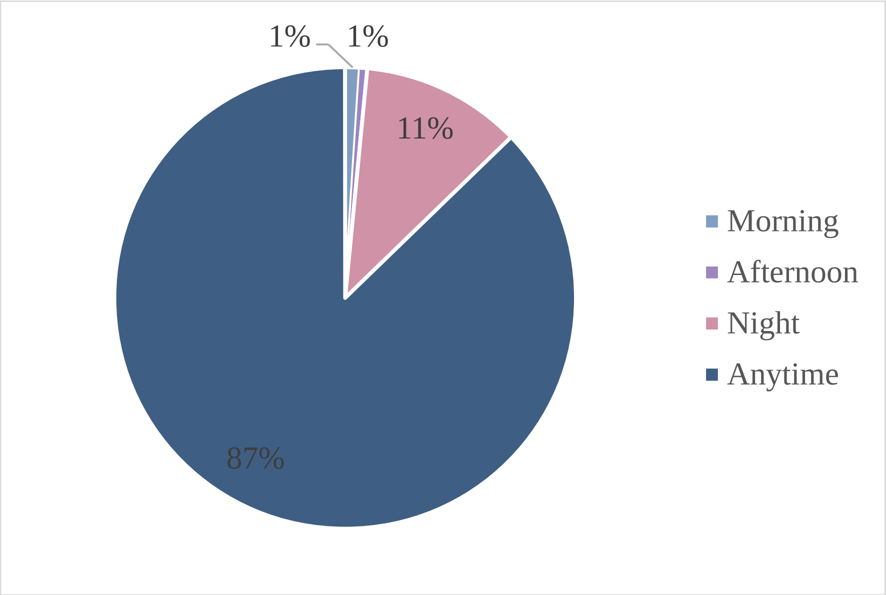

Of those randomised to m-follow up, 80.4% responded to at least one SMS, 54.2% responded to at least half the text messages sent but only 9.8% responded to all messages sent. 46.8% initiated some form of m-contact during the duration of follow up.

12.0 DISCUSSION

Ten-day adherence to prescribed zinc sulphate in children with AWD and no dehydration was high in the control group, which was not followed up by mobile. This was similar to the value found by researchers in Mali(24), but higher than that reported by researchers in Bangladesh(22) and India(23). This may be because of similarities in the two African populations and differences between the African and Asian populations studied. It may also have been higher in our study participants than in the Asian studies because of the adherence counselling provided by the recruiter while prescribing the medication.

There was no difference in adherence to zinc sulphate between the patients followed up via mobile phones and those followed up conventionally. The 10-day adherence for children followed up daily by mobile phone was slightly higher but this did not reach the targeted increase. The difference was also found to be of no statistical significance. This may have been due to the relatively high adherence in the population at baseline, making a significant increase impossible. Most studies showing an improvement in adherence to medication dealt with chronic illnesses where adherence is a bigger problem, affecting on average, over half of patients(41,42). This lower baseline adherence may leave more room for a significant improvement in adherence with various interventions, including mHealth practices.

Caregivers of the non-adherent children gave various reasons for their non-adherence (Figure 3 above). The three commonest reasons were directly or indirectly related to vomiting including stopping because of vomiting itself, a vomited dose not being repeated and running out of tablets before day 10 (because of repeating doses which were vomited) and not buying more to complete the prescribed duration . In total, two-thirds of non-adherence was associated with vomiting. This is in contrast to the study from Bangladesh (22) where vomiting accounted for only 12.2% of non-adherence, but where other factors such as the child being cured were more prominent. This may have been prevented by the adherence counselling provided to caregivers at presenting visit, emphasising that a full 10-day course be followed even if the child is feeling better.

The health facility used as a recruitment centre did not have zinc sulphate in stock. A few patients (all in the conventional follow-up arm) did not receive zinc sulphate from the pharmacies they visited. Non-adherence of this form may have been prevented had these

patients been followed up by mobile, since the parent/guardian would have been alerted to missing medication.

Children who presented with complaints of vomiting at enrolment were more than twice as likely to vomit their zinc sulphate as those with no vomiting at presenting visit. This points towards baseline nausea and vomiting associated with gastroenteritis playing a role in the vomiting of medication. The zinc sulphate prescribed may further aggravate this nausea and cause the vomiting, or it may play no specific role. **To the best of our knowledge, studies to look at the differences between rates of vomiting between different zinc salts have not been carried out yet. Similarly, the effect of different doses on vomiting and/or adherence has not been studied yet, but a large WHO trial is currently underway to determine the same. (ClinicalTrials.gov identifier** NCT03078842)

Of those meeting inclusion criteria, only two patients declined to participate in the study, showing the wide acceptability of m-follow up in our setting. Those who declined may have done so because they found m-follow up unacceptable or it may have been due to other reasons such as an inconvenience in filling out the picture diary, or coming back for a conventional follow-up visit on day 14.

Of those randomised to m-follow up, almost half initiated some form of m-contact during the duration of follow up. This was usually to ask for medical advice due to a change in the patient's condition such as development of dysentery or some dehydration. This shows that many patients, given the opportunity, would seek health-care/advice in a timely manner. This indicates that mHealth may improve health-seeking behaviour in those who are deterred by the cost of travelling to a health-care facility and/or time spent queuing to see a health-care provider.

A few patients could not be reached on the number(s) given at all times attempted – two were in the m-follow up group and seven in the control group. This disproportion may indicate a change in numbers between enrolment and attempted contact. If this is found to be the case upon further study, m-follow up may be less useful for longer term follow up.

These response rates are higher than those found in clients of TotoHealth Kenya where almost half never responded to SMSs and only a tenth responded often ***(Personal communication. F. Karim -- F. Ruiter, Totohealth. Aug 2016)***. This may have been because messages sent by TotoHealth are generally of an informative nature, seeking to educate about public health matters and remind of appointments when the child is usually well, whereas our messages were of a more probative nature when the child was unwell. Response rates in our study were higher with calls than text messages indicating that voice calls may be a more reliable method of contact.

12.1 STRENGTHS

There is high teledensity in Tanzania. This study was done in an urban setting where most of those assessed for eligibility owned a mobile phone, confirming that the teledensity in the study population was higher than the national average.

12.2 LIMITATIONS

We only enrolled children with AWD and no dehydration; therefore, our results may not be generalizable to all children with diarrhoea. **We also relied on caregiver-reported adherence to zinc sulphate as being accurate and did not count pills at the day 14 clinic visit. If reports were not factual, this is another limitation of the study.**

13.0 CONCLUSION

Most children with AWD and no dehydration were fully adherent to a 10-day course of zinc sulphate and m-Follow up did not significantly improve this adherence. Two-thirds of non-adherence is related to vomiting of medication, which is significantly associated with vomiting at presenting visit and should therefore be taken into account when prescribing/dispensing medication and planning follow-up. M-follow up is widely acceptable and dependable in our setting, and as this study demonstrates, improves adherence to a conventional follow up visit.

14.0 RECOMMENDATIONS

Adherence counselling alone with no other interventions produces a high adherence rate. This should be routine practice for zinc sulphate prescribed for diarrhoea. The doctor and dispensing pharmacist can both do this to ensure that it has been done well.

Children who present with vomiting at initial visit may be given a few extra zinc sulphate tablets to ensure adherence to the full 10-day course of zinc. They may also be followed up more closely (by mobile or physically) to ensure adherence. More studies are warranted to see if different zinc salts and/or different doses of zinc are better tolerated in those who present with vomiting at first encounter with the health care system.

M-follow up should be considered a viable option for improving attendance at follow-up visits. It does not significantly improve adherence to zinc sulphate, due to the good adherence even without other interventions; however, further studies need to be done to determine if it does, indeed, improve adherence to other prescribed short-term medications or not.

15.0 REFERENCES

1. WHO. Diarrhoeal disease. WHO factsheets. 2013.

2. Ne S, Haukenes G, Am S, Mhalu F. Rotavirus infection in Tanzania : a virological , epidemiological and clinical study among young children . APMIS acta Pathol Microbiol Immunol Scand. 1992;100(9):790–6.

3. Kb G, Aboud S, Cm M, Moyo S, Khavari N, Manji K, et al. Etiology of Diarrhea, Nutritional Outcomes and Novel Intestinal Biomarkers in Tanzanian Infants:A Preliminary Study. J Pediatr Gastroenterol Nutr. 2017;64(1):104–8.

4. De Vries TPGM, Henning RH, Hogerzeil HV, Fresle DA. Guide to Good Prescribing. WHO. 1994;

5. Garg M, Vishwakarma P, Sharma M, Nehra R, Saxena KK. The impact of irrational practices : A wake up call. J Pharmacol Phamacotherapeutics. 2014;5(4):245–7.

6. Rahman AE, Molla M, Worku A, Hurt L, Kirkwood B, Mohan SB, et al. Childhood diarrhoeal deaths in seven low- and middle-income countries. Bull World Health Organ. 2014;92(May):664–71.

7. Kanté AM, Gutierrez HR, Larsen AM, Jackson EF, Helleringer S, Exavery A, et al. Childhood Illness Prevalence and Health Seeking Behavior Patterns in Rural Tanzania. BMC Public Health [Internet]. BMC Public Health; 2015;15(1):951. Available from: http://dx.doi.org/10.1186/s12889-015-2264-6

8. Mashoto KO, Malebo HM, Msisiri E, Peter E. Prevalence , one week incidence and knowledge on causes of diarrhea: household survey of under-fives and adults in Mkuranga district, Tanzania. BMC Public Health. 2014;14:985.

9. Principal Secretary, MIS/Research data section, Welfare. M of H and S. Taarifa ya Magonjwa ya Kuhara (DTC): Dar 2014. (communicated 15/10/2015).

10. Hirschhorn N. The treatment of acute diarrhea in children: An historical and physiological perspective. Am J Clin Nutr. 1980;33(3):637–63.

11. WHO. WHO Drug Information: New ORS. World Health Organization. 2002. p. 121–2. Available from: http://apps.who.int/medicinedocs/pdf/s4950e/s4950e.pdf. Accessed 27/10/2017

12. Baqui AH, Black RE, Arifeen S El, Yunus M, Zaman K, Begum N, et al. Zinc therapy for diarrhoea increased the use of oral rehydration therapy and reduced the use of antibiotics in Bangladeshi children. J Heal Popul Nutr. 2004;22(4):440–2.

13. Lazzerini M, Ronfani L. Oral zinc for treating diarrhoea in children (Review). Cochrane Libr. 2013;(1).

14. Faruque ASG, Mahalanabis D, Haque SS, Fuchs GJ, Habte D. Double-blind , randomized , controlled trial of zinc or vitamin A supplementation in young children with acute diarrhoea. Acta Paediatr. 1999;88(c):154–60.

15. Bajait C, Thawani V. Role of zinc in pediatric diarrhea. Indian J Pharmacol. 2011;43(3):232–5.

16. Khan WU, Sellen DW. Zinc supplementation in the management of diarrhoea: Biological, behavioural and contextual rationale. Geneva, Switzerland WHO http//www.who.int/elena/titles/bbc/zinc_diarrhoea/en/. Published 2011. Accessed 15/01/2016.

17. Bhandari N, Bahl R, Taneja S, Strand T, Mølbak K, Ulvik RJ, et al. Substantial Reduction in Severe Diarrheal Morbidity by Daily Zinc Supplementation in Young North Indian Children. Pediatrics. 2002;109(6):e86.

18. Larson CP, Nasrin D, Saha A, Chowdhury MI, Qadri F. The added benefit of zinc supplementation after zinc treatment of acute childhood diarrhoea: a randomized , double-blind field trial. Trop Med Int Heal. 2010;15(6):754–61.

19. Mcdonald CM, Manji KP, Kisenge R, Aboud S, Spiegelman D, Fawzi WW, et al. Daily Zinc but Not Multivitamin Supplementation Reduces Diarrhea and Upper Respiratory Infections in Tanzanian Infants : A. J Nutr. 2015;(2):2153–60.

20. WHO. Implementing the New Recommendations on the Clinical Management of Diarrhoea Guidelines for Policy Makers and Programme Managers. 2006. 9 p.

21. Ministry of Health and Social Welfare. Tanzania National eHealth Strategy. 2013.

22. Nasrin D, Larson CP, Sultana S, Khan TU. Acceptability of and Adherence to Dispersible Zinc Tablet in the Treatment of Acute Childhood Diarrhoea. J Heal Popul Nutr. 2005;23(3):215–21.

23. Lamberti LM, Christa L, Walker F, Taneja S, Mazumder S, Robert E. Adherence to zinc supplementation guidelines for the treatment of diarrhea among children under – five in Uttar Pradesh , India. J Glob Health. 2015;5(2).

24. Winch PJ, Gilroy KE, Doumbia S, Patterson AMYE, Daou Z, Coulibaly S, et al. Short Report : Prescription and Administration of a 14-Day Regimen of Zinc Treatment for Childhood Diarrhea in Mali. Am J Trop Med Hyg. 2006;74(5):880–3.

25. Fukuoka Y, Vittinghoff E, Jong SS, Haskell W. Innovation to Motivation - Pilot study of a mobile phone intervention to increase physical activity among sedentary women. Prev Med (Baltim). 2011;51(3):287–9.

26. Shaw R, Bosworth H. Short message service (SMS) text messaging as an intervention medium for weight loss: A literature review. Health Informatics J. 2013;18(4):235–50.

27. Vervloet M, Linn AJ, Weert JCM Van, Bakker DH De, Bouvy ML, Dijk L Van. The effectiveness of interventions using electronic reminders to improve adherence to chronic medication : a systematic review of the literature. J Am Med Informatics Assoc. 2012;19(5):696–704.

28. Chande VT, Exum V. Follow-up Phone Calls After an Emergency Department Visit. Pediatrics. 1994;93(3):513–4.

29. Lin H, Chen W, Luo L, Congdon N, Zhang X, Zhong X, et al. Effectiveness of a short message reminder in increasing compliance with pediatric cataract treatment:

A randomized trial. Ophthalmology. 2012;119(12):2463–70.

30. Cormick G, Ciganda A, Cafferata ML, Ripple MJ, Estani SS, Buekens P, et al. Text message interventions for follow up of infants born to mothers positive for Chagas disease in Tucumán , Argentina : a feasibility study. BMC Res Notes. BioMed Central; 2015;8(1):508.

31. Pop-Eleches C, Thirumurthy H, Habyarimanae JP, Zivin JG, Goldstein MP, De Walque D, et al. Mobile phone technologies improve adherence to antiretroviral treatment in a resource-limited setting: a randomized controlled trial of text message reminders. AIDS. 2013;25(6):825–34.

32. House DR, Cheptinga P, Rusyniak DE. Availability of mobile phones for discharge follow-up of pediatric Emergency Department patients in western Kenya Study design. PeerJ. 2015;(3):e790.

33. Lund S, Hemed M, Nielsen BB, Said A, Said K, Makungu MH, et al. Mobile phones as a health communication tool to improve skilled attendance at delivery in Zanzibar: A cluster-randomised controlled trial. BJOG An Int J Obstet Gynaecol. 2012;119(10):1256–64.

34. Lund S, Rasch V, Hemed M, Boas IM, Said A, Said K, et al. Mobile phone intervention reduces perinatal mortality in zanzibar: secondary outcomes of a cluster randomized controlled trial. JMIR mHealth uHealth [Internet]. JMIR mHealth and uHealth; 2014 Jan 26 [cited 2016 Sep 1];2(1):e15. Available from: http://mhealth.jmir.org/2014/1/e15/

35. Lund S, Nielsen BB, Hemed M, Boas IM, Said A, Said K, et al. Mobile phones improve antenatal care attendance in Zanzibar: a cluster randomized controlled trial. BMC Pregnancy Childbirth. 2014;14(1):29.

36. Braun R, Lasway C, Agarwal S, L'Engle K, Layer E, Silas L, et al. An evaluation of a family planning mobile job aid for community health workers in Tanzania. Contraception. 2015;94(1):2–3.

37. Mitchell M, Hedt-Gauthier BL, Msellemu D, Nkaka M, Lesh N. Using electronic technology to improve clinical care: results from a before-after cluster trial to evaluate assessment and classification of sick children according to Integrated Management of Childhood Illness (IMCI) protocol in Tanzania. BMC Med Inform Decis Mak. 2013;13(1):1.

38. National Bureau of Statistics T. Sub-Divisional Population Projection for Year 2016 and 2017 Based on 2012 Population and Housing Census.

39. Temeke Municipal Council. The Image of Temeke. http://www.temekemc.go.tz/. p. http://www.temekemc.go.tz/storage/app/uploads/publ.

40. Zhong B. How to Calculate Sample Size in Randomized Controlled Trial ? J Thorac Dis. 2009;1(1):51–4.

41. Sabaté E. Adherence To Long-Term Therapies. World Heal Organ. 2003;7.

42. Osterberg L, Blaschke T. Adherence to Medication. N Engl J Med. 2005;353(5):487–97.

16.0 APPENDICES

16.1 APPENDIX 1: QUESTIONNAIRE

16.2 APPENDIX 2: PICTURE DIARY

16.3 APPENDIX 3: CONSENT FORM
